# How (not) to increase older adults’ tendency to anthropomorphise in serious games

**DOI:** 10.1371/journal.pone.0199948

**Published:** 2018-07-10

**Authors:** Barbara C. N. Müller, Shengnan Chen, Sari R. R. Nijssen, Simone Kühn

**Affiliations:** 1 Behavioural Science Institute, Radboud University, Nijmegen, Netherlands; 2 Klinik und Poliklinik für Psychiatrie und Psychotherapie, Universitätsklinikum Hamburg-Eppendorf, Hamburg, Germany; University of Minnesota, UNITED STATES

## Abstract

Among elderly, the use of serious games steadily increases. Research shows that anthropomorphising digital agents (i.e., ascribing human characteristics to them) has positive short-term consequences on interactions with digital agents. However, whether these effects can also be observed over a long-term period and in a real-life setting is unknown. In two studies, we investigated the important long-term consequences of anthropomorphism among older adults (age > 50) to increase involvement in serious games. Participants read either a story that highly anthropomorphized the digital agent of a training game, or a low anthropomorphism story about that agent. To investigate long-term effect, they played the training game for three weeks, and gaming data was assessed (number of games played, time of playing, points gained). While on the short-term, the anthropomorphic story increased the humanness of the agent (Study 1), no long-term effects where found (Study 2). Furthermore, an anthropomorphic story had no influence on the gaming outcome. Our results inform app developers about which techniques are useful to humanise digital agents.

## Introduction

The use of digital games increased steadily in the last decades, and also among older adults and the elderly, digital games become more and more common [1,2]. Serious gaming, that is, games that are designed to educate, as well as to entertain, is used as a way to teach people certain abilities and knowledge [3,4], train cognitive functioning [5–9], and improve (mental) health [10–12]. A common problem with serious games is that they tend to be less fun to play compared to commercial video games (i.e., games that are *not* designed to educate, next to entertain), and are therefore less effective in motivating players to reach specific learning goals. Therefore, in the present research, we investigated methods that can be used to increase player motivation in a serious game.

Interestingly, it seems that the more engaged players are with the game, the greater their improvement in learning [13,14]. Often, digital agents with a human-like appearance are implemented in games to help the player when necessary, and to engage the player. As these helping agents can increase the perceived usability of the game [15], and thus increase the player’s motivation, it is important to find additional ways to improve the interaction with such agents with methods that can easily be implemented in games. Research shows that one successful strategy is to provide a story describing the agent in a human-like way [16,17]. Whereas these studies only tested the immediate short-term effects (i.e., measuring the effects directly after providing the story) on interaction with digital agents, the present research investigates whether telling a human-like story also has long-term effects (i.e., measuring the effects three weeks after providing the story manipulation) on interactions with digital agents. These long-term, real-life effects are especially important when it comes to applying such top-down manipulations in applied settings or the introduction of digital agents in mobile applications. While it has been shown that these effects exist in a lab environment which is typically highly controlled with few distractions, the question remains whether providing a story describing a digital agent as a human-like being is enough to indeed increase the perceived humanness of this agent on a longer time scale and when daily-life distractions are present.

Research shows that people have the tendency to attribute human-like emotions, intentions, motivations, or characteristics to non-human agents and computers in order to establish a connection with them [18–24], a phenomenon called anthropomorphism. Seemingly, when people anthropomorphise, they generalise their knowledge about humans to nonhumans, implying that the same mental processes are involved in thinking about digital agents and humans [25,26]. For example, it has been shown that car fronts are perceived by recruiting brain regions that are specific for the processing of human faces [14], robots acting playfully are rated as being more extroverted than robots acting more seriously [27,28], and people apply stereotypes to robots and computers [29–31].

Researchers have started to explore how robots could be used to reduce loneliness among older adults [32]. Several studies confirm this link between anthropomorphism and social connection, demonstrating for example that people who anthropomorphised Pinocchio subsequently sat closer to a puppet of Pinocchio than people who did not anthropomorphise Pinocchio [33]. When reminded of close social bonds, people anthropomorphise less [34], and loneliness levels of elderly decrease with the company of a robot pet [35,36]. When anthropomorphism increases, people feel more understood by computer programs and more connected to them [37]. In line with this, trust in sophisticated technology (i.e., an autonomous driving car) increases when anthropomorphism increases [38], and credibility in robots improves when anthropomorphic cues are present [39]. In addition, digital agents which are rated more human-like are seen as more trustworthy, competent, sensitive, and warm [40], and anthropomorphism can increase trust resilience [41]. An increase in anthropomorphism can improve joint action situations [16, 42]: only when people were given the possibility to ascribe agency and intentions to a non-human agent, they started predicting this agent’s actions in a subsequent task, which is the foundation of any social interaction [43].

To be able to apply the positive consequences of anthropomorphism, it is therefore necessary to manipulate the humanness of a digital agent. Research shows that similarity with humans can be increased in two ways: first bottom-up by changing perceptual input, for example by increasing the perceptual similarity with humans [37, 44–47], make non-human agents move in a more human-like way [48], or program them in such a way that they show human emotions [49]. Second, top-down attributions increase anthropomorphism by making people ascribe agency, affect, or intentionality to the non-human agent [16, 42]. Research describing the effects of top-down attributions only investigated the short-term influence of top-down anthropomorphism. However, in an applied context, long-term effects are much more relevant: people do not just interact with a digital agent on their phone once for a few minutes, but continuously see it and interact with it on a daily basis. Yet, it is unknown whether the same effects of ascribing human-like characteristics that were found in short-term experimental studies also hold on the long-term. It would therefore be interesting to find an easy and low-cost way to increase top-down anthropomorphism towards digital agents on the long term. By doing so, engagement during game playing could be increased, and effectiveness of serious games could be improved.

In the present paper, we investigated whether providing a story about a non-human agent can effectively elicit long-term increase in anthropomorphism in older adults. Furthermore, we examined whether these possible changes have an influence on older adults’ motivation to play the game, measured by how often, how long, and how well they perform in a cognitive training game, the EinsteinTM Brain Trainer HD [50] which contains two digital agents, Einstein and Robo, a talking robot. As digital games are more and more often used in older adults and the elderly [1,2], for example to train cognitive functioning [6], our sample consisted of adults above 50 years of age.

To replicate earlier findings on the short-term effects in young adults [17, 42], in Study 1, older adults read a story about a digital agent which was either described in a highly human-like way (i.e., emphasising that the agent has intentions and emotions), or in a less human-like way (i.e., emphasising that the agent is programmed and is only performing pre-determined behaviours). Subsequently, participants’ tendency to anthropomorphise the agent, as well as their identification, and self-other overlap was assessed. In Study 2, long-term effects of the same stories were assessed over a period of three weeks, in which older adults were asked to play a cognitive training game. At the end of this period, the same evaluation questions were assessed. Based on earlier research [17], we assumed that while describing a non-human agent in a human-like way is a good means to increase anthropomorphism. Furthermore, we explore whether these effects are only working on the short-term or will be strong enough to persist long-term. Importantly, the goal of our research is to focus on how to increase anthropomorphism, not to investigate whether the game used is indeed helpful to increase cognitive performance.

## Study 1

### Methods

#### Participants and design

A power analysis indicated that a minimum of 38 participants would be required for achieving a statistical power of (1- β) = .85, based on an estimated effect size Cohen’s d = .61 found in earlier research [33]. Fifty-seven older adults (*M* = 70.51, *SD* = 4.51, age range 65–88 years, 23 female) participated in this study. They were recruited via e-mail through an elderly participant pool of Radboud University. Written informed consent was obtained from all participants prior to their participation in the study. All participants were fluent in Dutch and did not get paid for their participation. Participants were randomly assigned to a high anthropomorphism or low anthropomorphism condition. The participants assigned to the two groups did not differ in age (*t*(56) = -0.63, *p* = .53) or gender (***X***^*2*^(1) = 0.23, *p* = .63).

#### Procedure and materials

Participants were sent an invitation to participate in this study via e-mail. By clicking on a link in the e-mail, participants were redirected to a survey recorded with Qualtrics [51]. On the starting page, participants were informed that the survey was part of the development of an app designed to train one’s cognitive abilities; that the app would feature a digital agent named “Einstein”, and that the developers were interested in users’ experience of this digital agent. After providing their informed consent, participants were presented with a description and an image of the digital agent “Einstein”. In the high anthropomorphism group, participants were presented with a highly anthropomorphic description of the agent in which its emotions and agency was emphasized (203 words). In contrast, in the low anthropomorphism group, the agent was described in a mechanical way by emphasizing that it was merely programmed (173 words; see S1 Text for the stories). Both groups saw an identical image of the digital agent. After confirming that they had comprehensively read the description of “Einstein”, participants were presented with the five-item Anthropomorphic Mental State Ratings measuring the extent to which they perceived the digital agent to have a mind of its own, intentions, free will, consciousness, and experience emotions (e.g., “While reading the story, I felt like Einstein had his own thoughts.”, Cronbach’s α = .96; [52]). Subsequently, participants completed a three-item identification scale which assessed how strongly they identified with the digital agent and felt similar to the agent (e.g., “I identified myself with Einstein.”, Cronbach’s α = .81; adapted from [53]), and the Inclusion of Other in the Self Scale which assessed how close people feel towards the digital agent (IOS, [54]). This scale measures how strong others are included in the self. Two pairs of circles that vary on the level of overlap were presented to the participants; one was introduced as representing the participant, the other representing the digital agent. There were six different variations, and the higher the score on the IOS scale, the closer participants felt toward the digital agent. The IOS scale was always presented last, and the order of presentation of the AMSR and agent identification scales was counterbalanced; the order of the items within each scale was randomized (see S2 Text for complete questionnaire). After completing all survey items, participants were asked to provide demographic information (age and gender) and thanked for their participation.

### Results and discussion

To investigate whether story version had an influence on the evaluation of the non-human agent in a short-term assessment, a 2-way (story version: high anthropomorphism vs. low anthropomorphism) between-subjects MANOVA was conducted, with the mean anthropomorphism score, the mean identification score, and the IOS score as dependent variables. This analysis revealed a significant effect for the mean anthropomorphism score, *F*(1,55) = 23.078, *p* < .001, η_p_^2^ = .296, and a significant effect for the mean identification score, *F*(1,55) = 9.167, *p* = .004, η_p_^2^ = .143. The effect on the IOS score was not significant, *F*(1,55) = 2.674, *p* = .108, η_p_^2^ = .046. For means, standard deviation, and score range see Table 1.

**Table 1:** Means, standard deviations, and score-range for the dependent variables of Study 1.

	High anthropomorphism	Low anthropomorphism
anthropomorphism	5.36 (1.18), 1–6.40	3.29 (1.94), 1–7
identification	4.86 (0.98), 3–6.67	3.84 (1.48), 1–6.33
IOS	3.37 (1.21), 1–5	2.80 (1.40), 1–7

Results suggest that overall, the anthropomorphism manipulation was effective in a short-term assessment: after reading a story which emphasised the human-likeness of a non-human agent, older adults anthropomorphised the agent more, meaning that they ascribed more emotions and consciousness to the agent, and additionally, identify more with the agent. This is largely in line with earlier research showing that giving adults a story to read in which emotions and cognitions of a non-biological agent are highlighted increases anthropomorphic perception when directly measured after the manipulation, which in turn has pronounced influence on later interaction and social behaviours [16].

Although numerically it seems that self-other overlap increases, this difference did not reach significance. The fact that the Inclusion of the Other in the Self score did not differ between conditions as in earlier research [33] could potentially be due to differences in the design: while in previous research participants were confronted with a movie in which the main protagonist behaved in a human-like way, older adults who participated in the current study just read the story about the appearance of the protagonist, with a small picture placed next to the story. This could in turn make it more difficult to perceive similarity between oneself and the agent, which may explain why the differences in self-other overlap did not reach significance.

## Study 2

In our second study, we investigated whether comparable effects can be found when assessing anthropomorphism not immediately after the manipulation, as done in earlier research [33], but after a longer period, that is, three weeks after the manipulation. Furthermore, we examined whether these possible changes have an influence on how often, how long, and how well older adults play a cognitive training game, the Einstein Brain Trainer HD [50]. In doing so, we aimed at determining whether providing a story which describes a digital agent in a human-like way can increase older adults’ motivation to play a serious game.

### Methods

#### Participants and design

A power analysis indicated that a minimum of 38 participants would be required for achieving a statistical power of (1- β) = .85, based on an estimated effect size Cohen’s d = .61 found in earlier research [33]. Forty-two older adults (*M* = 62.67, *SD* = 8.52, age range 50–82 years, 29 female) participated in this study in exchange for 10 euro. They were recruited with flyers and letters distributed at supermarkets, elderly apartments, a community event, and the elderly participant pool of from Radboud University. Written informed consent was obtained from all participants prior to their participation in the study. All participants were at least 50 years old at the start of the study and owned an android smartphone or tablet. They were all fluent in Dutch. Ten participants had a bachelor’s or master’s degree, the majority of participants had a vocational education background (*n* = 28), and four went to secondary school or below. The study had a 2 (story version: high anthropomorphism vs. low anthropomorphism) between-subjects design, with the gaming variables (i.e., sum of points gained per day, duration in seconds per day, and number of finished games) as dependent variables. Participants were randomly assigned to one of the two conditions and were paid 10 euro. Additionally, they were entered into a 100-euro lottery as compensation for participation. This research was approved by the Radboud University Nijmegen Ethics Committee of the Faculty of Social Science.

#### Procedure and materials

Participants could respond to the flyers and letters by calling or emailing one of the researchers and indicating their willingness for participation. In case they met the inclusion criteria (>50 years of age, owning an Android smart phone or tablet, normal or corrected-to-normal vision, no physical impairments, no current or past neurological or psychiatric disorders), a first meeting was scheduled. During this meeting, the study procedure was introduced. The participants were told that we wanted to study the use of a brain training app, and that therefore, the Einstein Brain Trainer HD [50] would be installed to their device, and had to be played on a daily basis for the coming three weeks. With this game, they could play training games which fall in the categories logic thinking, memory training, vision training, or calculation training. After this introduction, written informed consent was obtained from the participants and the baseline interview was conducted. Participants had to answer questions about socio-demographic information such as gender, age, marital status, living arrangement, education (secondary school or below, vocational education, university or above). Subsequently, the training game app which contained two digital agents, Einstein and Robo, was installed by the researcher. At the same time, participants read one of the two stories used in Study 1. The high anthropomorphism story group was given a description of the digital agent as having emotion and agency; the low anthropomorphism group was given a description of the digital agent Einstein as having no emotions and agency, and as merely programmed. Subsequently, the researcher introduced the app, and was adapted by its developer for this study. The training game was reprogrammed to automatically generate logfiles that recorded for each game the date and time, the name of the game, the type of the game (single game, daily test, or workout), whether the game was finished, sum of points gained per day, duration in seconds per day, sum of correct answers per day, and sum of wrong answers per day. At last, it was explained to the participants how they could send the data collected by the app to the research team.

After the first and second week, participants in both groups got reminders about the digital agent (i.e., high anthropomorphism group: “If you have questions about the games, you can also click on the bubble over the head of Einstein "Do you need some help?", and he will be happy to assist you.” (reminder after the first week of study); “We, including Einstein, are happy that you are training your brains” (reminder after the second week of study); low anthropomorphism group: “If you have questions about the games, you can also click on the bubble over the head of the character Einstein "Do you need some help?" to read the explanations predetermined by the program.” (reminder after the first week of study); “We are happy that you are training your brains” (reminder after the second week of study).

After three weeks, the same measures as in Study 1 were used. Participants first completed the five-item Anthropomorphic Mental State Ratings (Cronbach’s α = .81; [52]). Second, they completed the three-item identification scale (Cronbach’s α = .74). Two questions about how much they liked the game (e.g., “I enjoyed playing the game”, “I would like to keep playing this game”) were asked. In case participants selected a score of 5, 6, or 7, they were asked to answer an open sub-question (e.g., “If you selected 5, 6, or 7: which games did you enjoy?”). Third, they also completed the IOS scale. Fourth, a five-item questionnaire was completed assessing for how long they owned the device that they used for the study, what they used it for, the frequency of use, and how convenient and helpful they perceived the device. The question about what they used the device for was an open question, and we categorized the answers by whether they played other games on the device. All other questions could be answered on a 7-point Likert scale ranging from 1 = not at all, 7 = very much (see S3 Text for complete questionnaire). After completion of all questions, participants were thanked, debriefed, and paid.

### Statistical analysis

We conducted the statistical analysis with SPSS version 22 [55], R version 3.3.0 [56], and JASP 0.8 [57]. To investigate whether story version had an influence on the evaluation of the non-human agent on the long term, a MANOVA was conducted with story version (high anthropomorphism vs. low anthropomorphism) as between-subjects factor, and the mean anthropomorphism scores, mean identification scores, and the IOS score as dependent variables. For the gaming data, mixed-model analyses were applied to take into account individual and time-based variations. In our models, the independent variables were story version (high anthropomorphism vs. low anthropomorphism), variation across participants, variation across time, as well as variation in the effect of the story version on the dependent variables across time; the dependent variables were the gaming variables (i.e., number of finished games per day, sum points of each day, and duration in seconds per day). We identified missing values (meaning that participants did not play the game on that day) and replaced them with zero’s when appropriate. Because we demonstrated the game on the first day of the 3-week period, the game log data of the first day were not included in the final analysis. We used model diagnostics to examine whether the models violated the assumptions of normality of residuals, homoscedasticity, and linearity. The models violated all these assumptions, so we decided to use bootstrapping methods to estimate the significance of the main effect.

### Results

#### Socio-demographics and user statistics

We conducted Welch two-sample t-tests for the continuous control variable (age, health, time they owned the device, frequency of use, convenience of the device, helpfulness of the device, enjoyment of the game), and Pearson’s *x*^*2*^ tests for the categorical control variables (gender, education level, living arrangement, marital status, playing other games on the device). The participants randomly assigned to the two groups did not differ on these variables, for detailed t-, *x*^*2*^-, and p-values, see Table 2.

**Table 2:** t-values, x^2^-values, and p-values of the control variables of Study 2.

age	*t*(40) = -0.98	*p* = .33
gender	*x*^*2*^(1) = 0.45	*p* = .50
educational level	*x*^*2*^(2) = 2.74	*p* = .25
living arrangement	*x*^*2*^(2) = 1.12	*p* = .57
marital status	*x*^*2*^(2) = 3.65	*p* = .16
health	*t*(38) = 1.65	*p* = .11
time owning the device	*t*(34.1) = -0.80	*p* = .43
frequency use	*t*(39.9) = 1.92	*p* = .06
convenience device	*t*(37.8) = 1.85	*p* = .07
helpfulness device	*t*(37.6) = 1.15	*p* = .26
playing games	*x*^*2*^(1) = 0.395	*p* = .53
enjoyment of the game	*t*(37.26) = -0.90	*p* = .37

Most participants were living with their partners, friends or family, and eight lived alone. Twenty-six participants were married, ten were in a relationship, and six were single. One participant was excluded from the analyses because no game data was submitted at the end of the study. Twenty-two participants played the game daily, and the 19 participants who did not play the game on some days missed 3.89 days on average (*SD* = 3.23). On average, each participant finished 4.06 games per day (*SD* = 4.39), with participants scoring 3921.24 points (*SD* = 4030.66) per day. The mean duration the app was used each day was approximately 5 minutes (*M* = 299.40 seconds, *SD* = 304.43).

#### Anthropomorphism, identification, and IOS

To investigate whether story version had an influence on the evaluation of the non-human agent on the long-term, a 2-way (story version: high anthropomorphism vs. low anthropomorphism) between-subjects MANOVA was conducted, with the mean anthropomorphism score, the mean identification score, and the IOS score as dependent variables. This analysis revealed no significant effect for the mean anthropomorphism score, *F*(1,40) = 0.510, *p* = .479, no significant effect for the mean identification score, *F*(1,40) = 0.424, *p* = .519, and no significant effect for the IOS score, *F*(1,40) = 0.026, *p* = .873.

To further investigate these non-significant effects, Bayesian statistical inference was conducted [58]. For the current study, a Bayesian independent samples t-test was performed. This yielded a BF01 = 2.69 for the anthropomorphism score, BF01 = 2.79 for the mean identification score, and BF01 = 3.27 for the IOS score. According to the Kass and Raftery [59] classification scheme, these Bayes factors provide weak to moderate evidence that the anthropomorphism, identification, and IOS scores are the same for all participants, regardless of story version. For means, standard deviation, and score range see Table 3.

**Table 3:** Means, standard deviations, and score-range for all dependent variables of Study 2.

	High anthropomorphism	Low anthropomorphism
anthropomorphism	2.43 (1.31), 1–4	2.71 (1.28), 1–3.8
identification	3.10 (1.35), 1–5.67	3.35 (1.17), 1–5.67
IOS	2 (1), 1–4	1.95 (0.92), 1–4

#### Gaming variables

We built one mixed effect model for each dependent variable, namely, the number of finished games, the duration of playing the game, and the performance in the game as reflected by the scores. One participant was excluded from further analysis because the game data was missing entirely. We investigated the effect of story version (sum-to-zero coded, with high anthropomorphism group as 1 and low anthropomorphism group as -1) on the dependent variable (fixed effects). To control for the individual and time-based variations, we modeled the variations in means around the subjects and time points, as well as the differing slopes for the effect of the story version on the dependent variable over time (random slopes). We also included all the random correlations.

Specifically, we built maximal linear mixed effects models [60] with the lmer() function from the lme4 package (version 1.1.13; [61]). To compute the significance of the main effect of story version, we used the bootstrapped Likelihood Ratio Test (1000 simulations) in the function PBmodcomp() from the package pbkrtest (version 0.4–6; [62]). We computed the correlation between the observed dependent variable and the fitted dependent variable (*r*) and used that to calculate the effect size—the amount of variance of the dependent variable explained by the model (*r*^*2*^). See Table 4 for an overview of the three models tested.

**Table 4:** R code and model statistics for the mixed effects models. The r^2^ was for the whole model, and the rest of the statistics were for the fixed effect of story version.

Dependent variable	*r*^*2*^	*B*	*SE*	PBtest	*p*
Sum of points	Model: sum of points ~ story + (1 | id) + (1 | time)				
	.45	21.47	430.31	-0.03	1.00
Duration	Model: duration ~ story + (1 | id) + (1 | time)				
	.46	-6.76	32.65	0.01	0.93
Number of finished games	Model: number of finished games ~ story + (1 | id)				
	.44	-0.20	0.47	0.15	0.70

All three models did not converge initially. For the model with the sum of points as the dependent variable, the random correlation between the random slope of story and the random intercept of time was 1, and the former had a lower variance (3322 vs. 85419). To make the model more parsimonious, we removed the random slope of story version. The reduced model converged and explained 45.33% of the variance in the scores. We found that the story version did not significantly predict the sum of points, estimate (*SE*) = 21.47 (430.31), PBtest = -0.03, *p* = 1.00. For the model with duration as the dependent variable, we also removed the random slope of story version, because it had a correlation of 1 with the random intercept of time, and its variance was smaller than the variance of the random intercept of time (4.34 vs. 611.43). The reduced model converged and explained 46.05% of the variance in the duration. We did not find the story version to be a significant predictor of the duration of play estimate (*SE*) = -6.76 (32.65), PBtest = 0.01, *p* = 0.93. Finally, for the model with the number of finished games as the dependent variable, we removed the random intercept of time and the random slope of story version, because both had a very low variance (0.22 and 0.0006, respectively). The reduced model converged and explained 44.04% of the variance in the number of finished games. We did not find the story version to be a significant predictor of the number of finished games, estimate (*SE*) = -0.20 (0.47), PBtest = 0.15, *p* = .70.

## General discussion

In the present research, we investigated whether providing a story about a non-human agent can elicit long-term effects and thus be an effective mean to increase anthropomorphism in older adults. Results of the present studies showed that although story type effectively increased human-likeness on the short-term, thus when immediately measured after reading the anthropomorphic story (Study 1), describing the digital agent in a human-like way did not have an effect on the level of anthropomorphism after three weeks, nor on how much or well older adults played the game (Study 2).

Previous research has found that different descriptions of the same non-human agent successfully manipulated people’s beliefs about how human-like this agent was, and providing stories which highlight the intentions and emotions of a digital agent is a proven way to successfully increase perceived human-likeness of these agents [16,17]. Study 1 replicated these findings and showed that people who read the high anthropomorphism story anthropomorphized and liked the digital agent Einstein more than those who read the low anthropomorphism story. At the same time, we extend this line of research by providing support for the notion that older adults show similar effects than younger adults [20]. Comparable to the above-mentioned studies, the level of anthropomorphism was measured shortly after presenting the story of the digital agent.

As results of Study 2 did not show any differences based on the story participants read, it is possible that the three-week interval was too long for the participants to retain the impression of the story that they read in the beginning of the study. Although we had a one-sentence reminder about the human-like / non-human-like attributes of the digital agent, this reminder was perhaps not strong enough to provide a human-like impression. Unfortunately, we did not assess human-like perception immediately after reading the story in Study 2, so we cannot say for sure whether our sample shows similar short-term effects as found in Study 1. However, given that this anthropomorphising effect is regularly found in earlier research among adults [16] when directly measured after the manipulation, we do not see why our sample should have shown a different response. We therefore suggest that the effects of the manipulation we used here did not endure over a three-weeks period. In line with this, recent longitudinal findings on player-avatar interactions showed that a higher perceptual anthropomorphism did not influence these interactions [63], suggesting that also bottom-up anthropomorphism has no positive effects on the long run.

Interestingly, recent research provided evidence for possible negative effect of anthropomorphising a digital agent in the context of video games [64]: more human-likeness lead to less game enjoyment because of a decrease in felt autonomy. Students who played a videogame with a digital agent designed to help them during the game rated a game as less enjoyable and felt less autonomy when the digital agent possessed more human-like features. Our findings do not show comparable negative consequences of a helping human-like digital agent, which can have three reasons. Firstly, our participants did play the game for a period of three weeks and during their daily routine, compared to the research conducted by Kim and colleagues where participants played the game for several minutes in a laboratory setting. Secondly, Kim and colleagues relied on a bottom-up manipulation of anthropomorphism, using perceptual human-likeness instead of the here used top-down manipulation. Thirdly, the participants of our study were much older and might therefore be less experienced with playing games. Therefore, it might be possible that on the long run, the possible negative effects found on game enjoyment do not hold, can only be found for certain types of digital agents, or for certain, more experienced groups of players. This could imply that while less experienced players such as in the current study would be more satisfied with an anthropomorphic agent helping them, more experienced players would show a decrease in autonomy and gaming satisfaction. Future research is necessary to investigate these possible explanations.

The current study design could be improved by including another control group with the same game but without digital agents. By doing so, it is possible to investigate the effects of the mere presence of a digital agent and see whether this has an additional value to using a training game. Additionally, it would be interesting to investigate whether the perceptual human-likeness has an influence on playing the game; it might be possible that top-down manipulation of anthropomorphism with a story is possible in case the digital agent is not very human-like, for example a mechanical robot. In the present design, it could be that the perceptual humanness of the digital agent (and the fact that Einstein was a real human) was strong enough to induce anthropomorphism and no additional increase was possible. Related to this point, it should be investigated whether more lively manipulations (e.g., showing a video in which the avatar acts intentional [16]; presenting longer stories to increase perspective-taking [65]) lead to stronger effects, and are thus more effective in influencing anthropomorphism and related variables over a longer period of time. Lastly, given that all participants in our study were Dutch, a western society with relatively easy access to digital devices like computers or smart phones, results should be replicated in other cultures to see whether our findings are generalizable.

Because we conducted our study in a natural setting over three weeks, we could better understand the implications of interacting with technology in our daily lives. The present research raises the question of whether it is relevant and useful to add human-like features to digital agents in training games for older adults. Although the digital agent Einstein already has a human-like appearance, uses gestures, and speech, the participants did not tend to anthropomorphize him over a period of three weeks. And even the participants who stronger anthropomorphized Einstein did not play the game more often or performed better. Thus, our research revealed that although a simple story manipulation has beneficial short-term effects in a lab setting, leading to higher human-likeness, it does not lead to higher level of anthropomorphism among older adults after a period of three weeks. As technology is becoming more and more prominent in the care for older adults, our results can inform the designers about the usefulness of human-like attributes of digital agents.

## Supporting information

S1 Text English translation of both stories used in Study 1 and Study 2. (DOCX)

S2 Text English translation of questionnaire items Study 1. (DOCX)

S3 Text English translation of questionnaire items Study 2. (DOCX)
