# Supplementary material for: How (not) to increase older adults’ tendency to anthropomorphise in serious games
Source: PLoS One. 2018 Jul 10;13(7):e0199948. doi: 10.1371/journal.pone.0199948 (PMC6039013; doi:10.1371/journal.pone.0199948)
Supplement: S1 Text — (DOCX) [file pone.0199948.s001.docx]

**S1 text: English translation of both stories used in Study 1 and Study 2**

**High anthropomorphism story**

Einstein is very interested in helping people train their brains, so the Brain Trainer game invited him to be a consultant. In the game, he is wearing his favorite green woolen vest and of course he is sporting his iconic hairstyle. He looks like an authoritative figure, but he is friendly and patient. He likes helping and supporting people. He greets people every time they start the game and gives them instructions about the game. He is happy to answer any questions they may have. To him, the people playing the game are like his colleagues. When they do well in a game, he applauds them because he is pleased with their progress. When they do not train their brains for a while, he is disappointed and feels a bit alone. Besides advising people in the game, he is busy developing his latest theories at Princeton University. As a hobby, he also enjoys playing the violin. He likes to perform for small private audiences and friends. He likes to take long walks in the park, where reflects upon his life. He is happy with the fact that he has lived a fulfilling life.

**Low anthropomorphism story**

The Einstein character was designed by a game company. The company dressed him in a green woolen vest and gave him a hairstyle that resembles the real Einstein’s hairstyle. In order to make him look as realistic and helpful as possible, the developers programmed his facial expressions to change. Sometimes he looks like an authoritative figure, and sometimes he looks very friendly. The program determines that he appears every time the game is opened. The program has placed speech bubbles over his head with pre-determined text. Players can click on those speech bubbles to get more information about the game. In addition, an algorithm makes him applaud when a player scores points. He is basically a string of codes, which renders his appearance, actions, and expressions. By being programed like that, people have the illusion that the character Einstein is responding to their actions. When a player quits the game, Einstein no longer exists, just like an image on TV that disappears when the TV is turned off.
