# Supplementary material for: How (not) to increase older adults’ tendency to anthropomorphise in serious games
Source: PLoS One. 2018 Jul 10;13(7):e0199948. doi: 10.1371/journal.pone.0199948 (PMC6039013; doi:10.1371/journal.pone.0199948)

**S2 text: English translation of questionnaire items Study 1**

Circle the number that best represents your answer. Thereby, 1 stands for "not at all" and 7 stands for "very much". There are no right or wrong answers, therefore try to follow your feelings.

**Anthropomorphic Mental State Ratings**

1. While reading the story, I felt that Einstein had his own will.

1 ----- 2 ----- 3 ----- 4 ----- 5 ----- 6 ----- 7

not at all very much

1. While reading the story, I felt like Einstein acted according to his own intentions.

1 ----- 2 ----- 3 ----- 4 ----- 5 ----- 6 ----- 7

not at all very much

1. While reading the story, I felt like Einstein had a mind of his own.

1 ----- 2 ----- 3 ----- 4 ----- 5 ----- 6 ----- 7

not at all very much

1. While reading the story, I felt like Einstein experienced emotions.

1 ----- 2 ----- 3 ----- 4 ----- 5 ----- 6 ----- 7

not at all very much

1. While reading the story, I felt like Einstein had consciousness.

1 ----- 2 ----- 3 ----- 4 ----- 5 ----- 6 ----- 7

not at all very much

**Identification Scale**

1. I identified with Einstein.

1 ----- 2 ----- 3 ----- 4 ----- 5 ----- 6 ----- 7

not at all very much

1. I liked Einstein.

1 ----- 2 ----- 3 ----- 4 ----- 5 ----- 6 ----- 7

not at all very much

1. I enjoyed seeing Einstein.

1 ----- 2 ----- 3 ----- 4 ----- 5 ----- 6 ----- 7

not at all very much

**Inclusion of Other in the Self Scale**

Below are six images, each one featuring two circles. Imagine that you are one of the circles, and that the other circle represents Einstein. De images differ in how near or far the circles are to each other. Indicate, by choosing one of the six images, how close or far away you feel towards Einstein. You can answer by picking a number between 1-6.


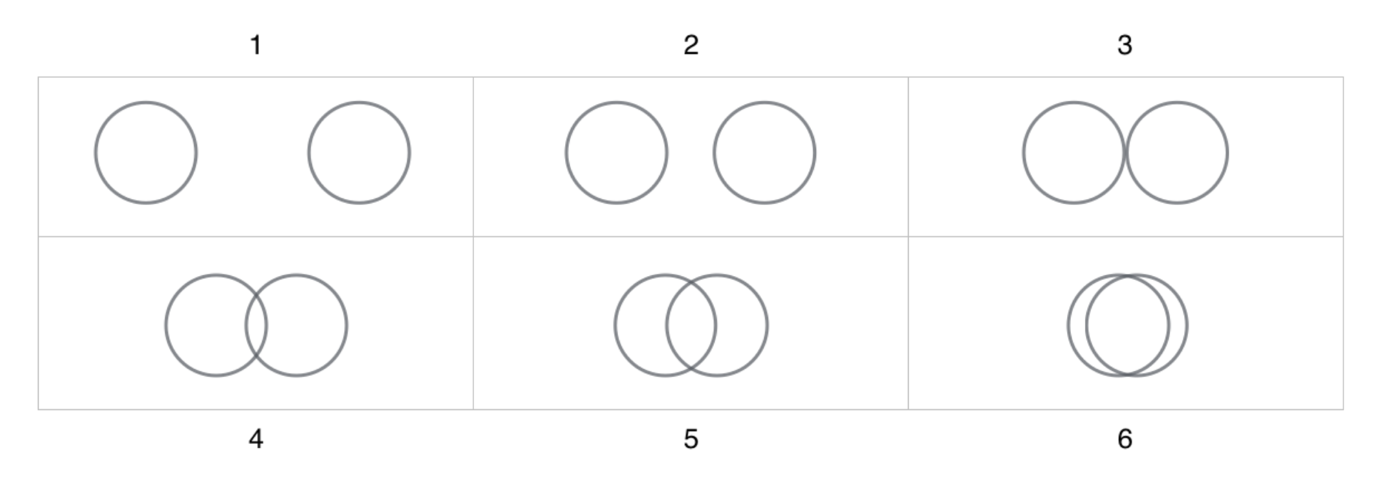

Supplement: S2 Text — (DOCX) [file pone.0199948.s002.docx]
