# Supplementary material for: How (not) to increase older adults’ tendency to anthropomorphise in serious games
Source: PLoS One. 2018 Jul 10;13(7):e0199948. doi: 10.1371/journal.pone.0199948 (PMC6039013; doi:10.1371/journal.pone.0199948)
Supplement: S3 Text — (DOCX) [file pone.0199948.s003.docx]

**S3 text: English translation of questionnaire items Study 2**

Circle the number that best represents your answer. Thereby, 1 stands for "not at all" and 7 stands for "very much". There are no right or wrong answers, therefore try to follow your feelings.

**Anthropomorphic Mental State Ratings**

1. While playing the game, I felt that Einstein had his own will.

1 ----- 2 ----- 3 ----- 4 ----- 5 ----- 6 ----- 7

not at all very much

1. While playing the game, I felt like Einstein acted according to his own intentions.

1 ----- 2 ----- 3 ----- 4 ----- 5 ----- 6 ----- 7

not at all very much

(3) While playing the game, I felt like Einstein had a mind of his own.

1 ----- 2 ----- 3 ----- 4 ----- 5 ----- 6 ----- 7

not at all very much

1. While playing the game, I felt like Einstein experienced emotions.

1 ----- 2 ----- 3 ----- 4 ----- 5 ----- 6 ----- 7

not at all very much

1. While playing the game, I felt like Einstein had consciousness.

1 ----- 2 ----- 3 ----- 4 ----- 5 ----- 6 ----- 7

not at all very much

**Identification Scale**

(1) I identified with Einstein.

1 ----- 2 ----- 3 ----- 4 ----- 5 ----- 6 ----- 7

not at all very much

(2) I liked Einstein.

1 ----- 2 ----- 3 ----- 4 ----- 5 ----- 6 ----- 7

not at all very much

(3) I enjoyed Einstein’s company in the game.

1 ----- 2 ----- 3 ----- 4 ----- 5 ----- 6 ----- 7

not at all very much

**Questions about the game**

1. I enjoyed playing the game.

1 ----- 2 ----- 3 ----- 4 ----- 5 ----- 6 ----- 7

not at all very much

If you answered 5, 6, or 7: which games did you like?

1. I would like to keep playing this game.

1 ----- 2 ----- 3 ----- 4 ----- 5 ----- 6 ----- 7

not at all very much

**Inclusion of Other in the Self Scale**

Below are six images, each one featuring two circles. Imagine that you are one of the circles, and that the other circle represents Einstein. De images differ in how near or far the circles are to each other. Indicate, by choosing one of the six images, how close or far away you feel towards Einstein. You can answer by picking a number between 1-6.


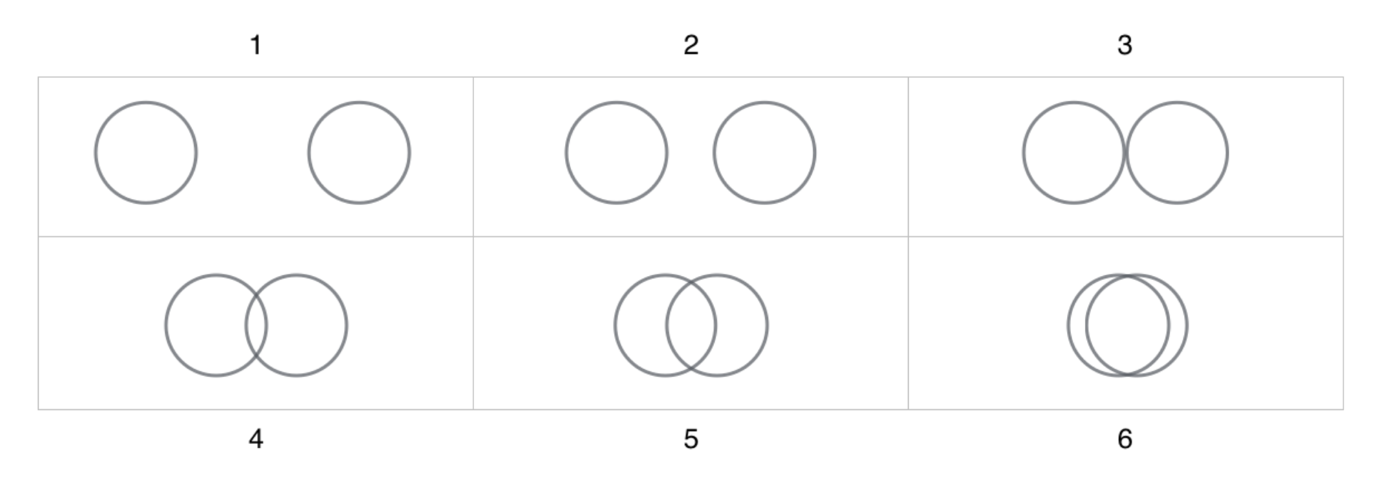


**Device usage**

We would like to know about your experience of using your smartphone or tablet (think of the one that you used in this study).

1. How long have you had this device? __ years __ months
2. For what do you use this device?
3. How often do you use your device (outside of this experiment)? Circle the number that best represents your answer.
4. less than once per day
5. once per day
6. two to five times per day
7. once per hour
8. more than once per hour
9. How convenient to use do you find this device? Circle the number that best represents your answer.

1 ----- 2 ----- 3 ----- 4 ----- 5 ----- 6 ----- 7

not at all very much

1. How helpful is this device for you in your daily life? Circle the number that best represents your answer.

1 ----- 2 ----- 3 ----- 4 ----- 5 ----- 6 ----- 7

not at all very much
